# LINC1467 Activates the IPO8–p65 Axis to Restrict Hand, Foot, and Mouth Disease Virus Replication

**DOI:** 10.3390/pathogens14101071

**Published:** 2025-10-21

**Authors:** Xiaokui Zhang, Jinwei Li, Li Ding, Jihong Zhang, Fan Yang, Yonghan Luo, Wei Chen

**Affiliations:** 1 Medical School, Kunming University of Science and Technology, Kunming 650500, China; zhangxiaokui@stu.kust.edu.cn (X.Z.);; 2 The First People’s Hospital of Yunnan Province, Kunming 650500, China; 3 Faculty of Life Science and Technology, Kunming University of Science and Technology, Kunming 650500, China

**Keywords:** long non-coding RNA (lncRNA) LINC1467, hand-foot-mouth-disease (HFMD) related human enteroviruses (EVs), p65 phosphorylation, pro-inflammatory cytokines, Importin 8 (IPO8)

## Abstract

Hand–foot–mouth disease (HFMD), primarily caused by human enteroviruses (EVs), poses a public health challenge, particularly among infants, due to a lack of effective therapies. Elucidating the molecular interplay between EVs and the host immune response is crucial for developing antiviral treatments. Recent studies have highlighted the significance of long non-coding RNAs (lncRNAs) in regulating host–pathogen interactions; however, the mechanisms of lncRNAs in EV infection remain poorly unexplored. Here, we identified a highly inducible nuclear lncRNA, LINC1467, that is upregulated in response to HFMD-related EV infection. Functional analyses revealed that LINC1467 suppresses viral replication. Mechanistically, LINC1467 interacts with nuclear import receptor Importin 8 (IPO8) to form the LINC1467/IPO8/p65 complex, facilitating the phosphorylation and nuclear translocation of p65, thus promoting the expression of pro-inflammatory cytokines and activating the NF-κB pathway. The antiviral function of LINC1467 was further validated in a mouse model of viral infection. These findings uncover a novel lncRNA-mediated regulatory mechanism in the innate immune response and highlight LINC1467 as a promising target for future antiviral strategies against HFMD-related EVs.

## 1. Introduction

Hand–foot–mouth disease (HFMD) is an acute infectious illness caused by human enteroviruses (EVs), primarily transmitted via the fecal–oral route. Clinically, HFMD is characterized by fever and vesicular eruptions on the hands, feet, and oral mucosa. Although most cases are self-limiting, severe complications such as aseptic meningitis, viral encephalitis, myocarditis, and acute flaccid paralysis can potentially result in death [1,2]. Epidemics or outbreaks of HFMD have been reported globally, with particularly severity in the Asia–Pacific region. HFMD poses a serious threat to infant health and represents a considerable global economic burden [3,4,5,6]. However, no effective treatments or vaccines are currently available. The primary etiological agents of HFMD—Enterovirus 71 (EV71), coxsackievirus A16 (CVA16), and coxsackievirus B5 (CVB5)—belong to the genus Enterovirus within the family Picornaviridae, and have a positive-sense single-stranded RNA virus. The viral genome is approximately 7.5 kb in size, encoding four structural proteins (VP1, VP2, VP3 and VP4) and seven non-structural proteins (2A, 2B, 2C, 3A, 3B, 3C and 3D). VP1 is the primary antigenic determinant and plays an important role in viral infection, while non-structural proteins are essential for genome replication and virus assembly [7].

Long noncoding RNAs (lncRNAs) are transcripts longer than 200 nucleotides. They are versatile molecules that can interact with DNA, RNA, and proteins, functioning as guides, scaffolds, sponges, and molecular decoys [8]. A growing body of research suggests that lncRNAs are key regulators of innate immunity against viral infections [9]. Nuclear factor kappa-B (NF-κB) is a master transcriptional regulator of inflammatory cytokines and antiviral genes in innate immunity. Emerging studies indicate that lncRNAs modulate the NF-κB signaling pathway [10]. For example, LncRNA-AK149641 binds to NF-κB in the nucleus, thereby decreasing TNF-α secretion to regulate the inflammatory response in mast cells [11]. LncRNA PILA leads to an increase in the transcription of the gene encoding transforming growth factor β-activated kinase 1 (TAK1), an upstream activator of NF-κB signaling, thereby enhancing the signaling pathway [12]. LncRNA PINT serves as a molecular scaffold linking p65 and enhancer of zeste homolog 2 (EZH2) to regulate the transcription of inflammatory factors, highlighting the importance of this lncRNA as a potential therapeutic target in infectious diseases [13]. Additionally, lncRNAs such as JINR1, which interacts with RBM10 to activate NF-κB during flavivirus infection, and LINC01197, which is upregulated in response to influenza A virus and modulated by NF-κB, underscore the broad role of lncRNAs in host–virus interactions [14,15].

In our previous work, we analyzed the lncRNA expression profiles of SH-SY5Y cells infected with CVB5 [16]. In our current research, we focus on a nuclear-enriched lncRNA, LINC1467, which is markedly upregulated upon infection with HFMD-related EVs. We demonstrate that LINC1467 inhibits the replication of HFMD-related EVs. Mechanistically, LINC1467 interacts with nuclear import receptor Importin 8 (IPO8), forming a LINC1467/IPO8/p65 complex that promotes p65 phosphorylation and nuclear translocation, thereby activating the NF-κB pathway and enhancing pro-inflammatory cytokine expression to exert their function. The antiviral role of LINC1467 was further validated in a mouse model of HFMD. Collectively, our findings identify LINC1467 as a novel positive regulator of the host antiviral response and suggest its potential as a therapeutic target for the development of new antiviral therapies.

## 2. Materials and Methods

### 2.1. Cells and Virus

All cell lines and viral strains were persevered in our laboratory. Human neuroblastoma (SH-SY5Y), human rhabdomyosarcoma (RD), human astrocytoma (U373 and U87), human glioma (U251), normal human intestinal epithelial (HIEC), human normal colon epithelial (CCD841), murine monocyte–macrophage leukemia (RAW264.7), and African green monkey kidney (Vero) cells were cultured in Dulbecco’s modified eagle’s medium (DMEM) (41500034, Servicebio, Wuhan, China) supplemented with 10% fetal bovine serum (FBS) (FBS-E500, NEWZERUM, Christchurch, New Zealand). Human monocytic leukemia (THP-1), human colon carcinoma (HT29), and murine colon carcinoma (CT26) were cultured in a DMEM/F12 medium (D6570, Servicebio, China) supplemented with 10% FBS. All cells were maintained at 37 °C in a humidified atmosphere with 5% CO_2_.

The CVB5 (GenBank: MH201081.1), EV71 (GenBank: MK028135.1) and CVA16 (GenBank: KY440934.1) strains were serially diluted 10^−1^–10^−12^ with DMEM containing 2% FBS. Vero cells were infected with serial dilutions and the cytopathic effect (CPE) was recorded 5–7 days post-infection. The 50% tissue culture infectious dose (TCID_50_) was calculated using the Reed–Muench method.

### 2.2. Plasmids and Transfection

The full-length sequence of LINC1467 was cloned into the pcDNA3.1(+) vector for overexpression. To specifically knockdown nuclear-localized LINC1467, antisense oligonucleotides (ASOs) with locked nucleic acid (LNA) modifications were performed. The following ASO duplexes were synthesized by Guangzhou RiboBio Co., Ltd. (Guangzhou, China): ASO-LINC1467-1: 5′-CTTCAAATCGGGATGCAAA-3′; ASO-LINC1467-2: 5′-TTCAATTCGGGATGCAAAC-3′; ASO-LINC1467-3: 5′-AATCGGGATGCAAACTTCA-3′ and the negative control (NC). Cells were transfected with plasmids using Lipofectamine 3000 (L3000008, Thermo, Waltham, MA, USA) for 24 h according to the manufacturer’s instructions.

### 2.3. RNA Extraction and Real-Time Quantitative PCR (RT-qPCR)

Total RNA was extracted from transfected cells using RNAiso Plus (9108, Takara, Kyoto, Japan). Reverse transcription was performed with Hifair^®^ II 1st Strand cDNA Synthesis SuperMix for qPCR (11123ES60, Yeasen, China). qPCR was conducted using Hieff^®^ qPCR SYBR Green Master Mix (11123ES03, Yeasen, Shanghai, China) on a 7500 Real-Time PCR System. The cycling conditions were as follows: 95 °C for 5 min, followed by 40 cycles of 95 °C for 5 s and 60 °C for 34 s. Relative mRNA levels were calculated using the 2 ^−ΔΔCt^ method and normalized to GAPDH. All primer sequences are listed in Table 1.

### 2.4. Immunoprecipitation and Western Blotting

Cells were lysed in immunoprecipitation buffer, and incubated with protein A/G magnetic beads and antibodies. After washing, the immunoprecipitated protein complexes were eluted, denatured and mixed with the loading buffer, followed by boiling for Western blotting analysis.

Proteins were separated by sodium dodecyl sulfate–polyacrylamide gel electrophoresis (SDS-PAGE) and subsequently transferred onto polyvinylidene difluoride filter (PVDF) membranes. Membranes were blocked with 5% (*w*/*v*) skimmed milk for 2 h at room temperature, incubated with primary antibodies overnight at 4 °C. After washing three times with Tris-buffered saline containing 0.05% Tween 20 (TBST), the membranes were incubated with secondary antibodies for 1 h at room temperature. Protein bands were visualized using an enhanced chemiluminescence detection system.

### 2.5. RNA Immunoprecipitation (RIP) Assay

RIP assays were performed using the Dynabeads™ Protein A Immunoprecipitation Kit (10006D, Thermo, USA) following the manufacturer’s instructions. Briefly, cells were collected and lysed with immunoprecipitation buffer. RNA–protein complexes were immunoprecipitated with antibody–conjugated protein A/G beads. After washing, the RNA–binding proteins were purified. The enrichment of specific RNA was quantified by qPCR and normalized to the input RNA.

### 2.6. RNA Pull-Down Assay

Biotin-labeled LINC1467 RNA was transcribed with MEGAscript™ T7 Kit (AM1333, Thermo, USA) and purified with Monarch^®^ RNA Cleanup Kit (T2050, New England Biolabs). The biotin-labeled antisense strand of LINC1467 served as a negative control. RNA–protein complexes were conducted using the Pierce™ Magnetic RNA-Protein Pull-Down Kit (#20164, Thermo, USA). After washing and elution, the bead–biotin RNA–protein complex was analyzed for Western blotting.

### 2.7. Cytoplasmic and Nuclear Fractionation

Cells were fractionated using the PARIS Kit (AM1921, Invitrogen, Carlsbad, CA, USA). Cytoplasmic and nuclear fractions were separated by centrifugation, and the nuclear pellets subsequently lysed. RNA was extracted using a lysis/binding solution, followed by qPCR to analysis. GAPDH and U6 were used as cytoplasmic and nuclear reference genes, respectively.

### 2.8. Enzyme-Linked Immunosorbent Assay (ELISA)

Cells were transfected with plasmids, and the culture supernatant was collected to quantify cytokine production. The concentrations of human TNF-α (MM-0122H1), IL-1β (MM-0181H1), IL-6 (MM-0049H1), and CCL5 (MM-14376H1) levels were measured by ELISA kits (MEIMIAN) according to the manufacturer’s instructions.

### 2.9. Mouse Model

BALB/c mice (6–8 weeks old) were obtained from Yunnan University (Kunming, China) and housed in a specific pathogen-free (SPF) facility at the Experimental Animal Center of Kunming University of Science and Technology. All animal procedures were approved by the Institutional Animal Care and Treatment Committee (no. PZWH (Dian) K2023-0044).

LINC1467 overexpression plasmid and a control vector were packaged into an adeno-associated virus (AAV9) with the fluorescent reporter (NewHelix Biotech, Shanghai, China). Three-day-old BALB/c mice were intraperitoneally injected with fresh AAV vectors (5 × 10^11^ genomic copies). Fourteen days post-infection, the mice were intraperitoneally challenged with CVB5 or EV71 (20 LD_50_), and their health status was monitored daily.

### 2.10. Hematoxylin–Eosin (HE) Staining

Tissue samples were fixed in 4% paraformaldehyde at 4 °C for 24 h, dehydrated through a graded ethanol series, and embedded in paraffin. Paraffin-embedded tissue sections were deparaffinized, rehydrated, and stained with hematoxylin and eosin (HE) using standard protocols. After dehydration and clearing, sections were mounted and examined under a light microscope.

### 2.11. Statistical Analysis

Data are presented as the mean ± standard deviation (SD) with an error bar that represents at least three independent experiments. Statistical significance was evaluated using a two-tailed Student’s *t*-test in GraphPad Prism (version 9.0). A *p*-value of less than 0.05 was considered statistically significant (* *p* ≤ 0.05, ** *p* ≤ 0.01 and *** *p* ≤ 0.001).

## 3. Result

### 3.1. Characterization of LINC1467 in SH-SY5Y Cells

**Figure 1:**
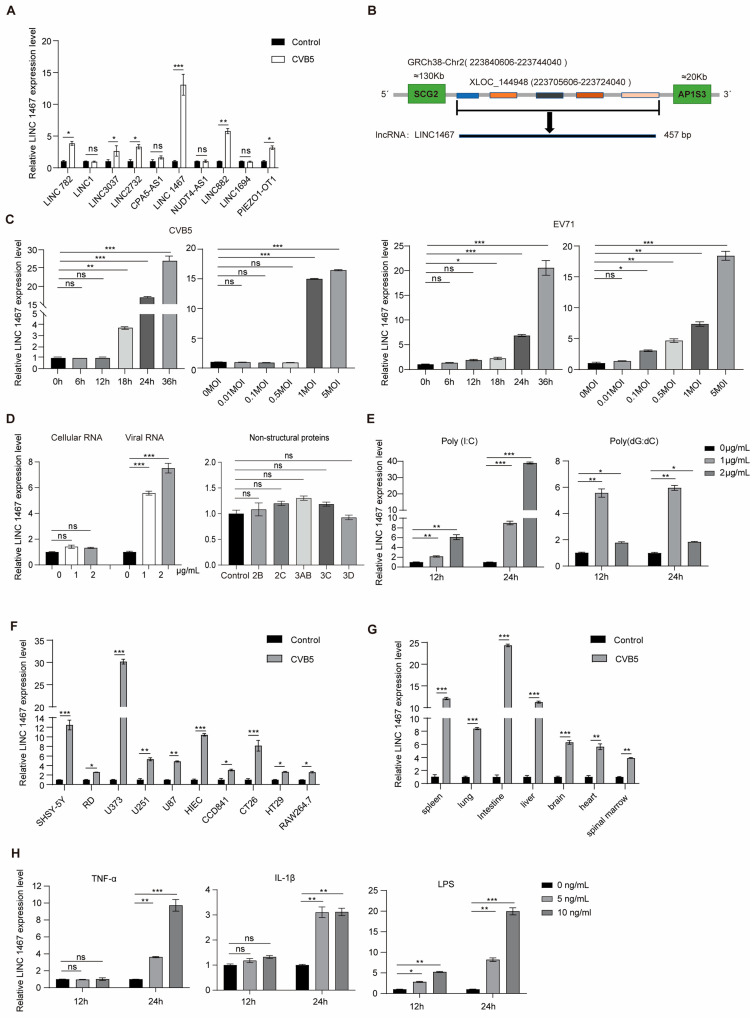
Identification and characterization of LINC1467 in SH-SY5Y cells. (**A**) SH-SY5Y cells were infected with CVB5, followed by RT-qPCR validation of the top 10 differentially expressed lncRNAs related to inflammation. (**B**) The location of human LINC1467 in the genome. (**C**) SH-SY5Y cells were infected with CVB5 or EV71 at varying MOIs or for the indicated time intervals. The expression of LINC1467 was measured by RT-qPCR; (**D**) SH-SY5Y cells were transfected with increasing amounts of CVB5 RNA, or with plasmids (2 μg) encoding viral nonstructural proteins. The expression of LINC1467 was measured by RT-qPCR; (**E**) SH-SY5Y cells were stimulated with different amounts of poly (I:C) or poly (dG:dC) for 24 h. The expression of LINC1467 was measured by RT-qPCR; (**F**) multiple cell lines were infected with CVB5 (MOI = 1) for 24 h. The expression of LINC1467 was measured by RT-qPCR; (**G**) three-day-old BALB/c mice were infected with CVB5 (20 LD50) for 10 days. The expression of LINC1467 was measured by RT-qPCR. (**H**) SH-SY5Y cells were treated with increasing amounts of TNF-α, IL-1β and LPS for 12 h and 24 h, respectively. The expression of LINC1467 was measured by RT-qPCR. All data are presented as the mean ± SD from biologically independent experiments (*n* = 3). Significant analysis was performed using Student’s *t*-test: *p* ≤ 0.05 (*), *p* ≤ 0.01 (**), *p* ≤ 0.001 (***), and ns for no significant difference.

To identify the host lncRNAs involved in HFMD-related EV infection, we analyzed the transcriptomes of CVB5-infected SH-SY5Y cells via RNA sequencing (RNA-seq) and identified the top differentially expressed lncRNAs (Figure S1). RT-qPCR validation confirmed that the level of LINC1467 was significantly upregulated in SH-SY5Y cells following CVB5 infection (Figure 1A). LINC1467 is a 457 bp intergenic lncRNA located on chromosome 2 (Figure 1B). PhyloCSF analysis indicated that LINC1467 lacks protein-coding potential (PhyloCSF score < 0) (Figure S2). Cross-species sequences alignment across 13 representative species (UCSC Genome Browser) showed that the high evolutionary conservation of LINC1467 (Figure S3).

We next examined the dynamics of LINC1467 during CVB5 and EV71 infection. The results showed that the expression of LINC1467 exhibited both a dose- and time-dependent manner upon CVB5 infection, and a similar induction pattern was observed during EV71 infection (Figure 1C). To determine the viral components responsible for the induction, SH-SY5Y cells were transfected with the CVB5 genome and non-structural proteins. Results revealed that the expression of LINC1467 was specifically induced by the viral genome, while non-structural proteins had no effect (Figure 1D). The double-stranded RNA mimic poly(I:C) and the DNA mimic poly(dG:dC) confirmed the results (Figure 1E). Moreover, the expression of LINC1467 was significantly upregulated in a range of CVB5-infected cell lines, indicating its broad induction across diverse cell types (Figure 1F). Consistent with the in vitro results, the expression of LINC1467 was highly expressed in various tissues of CVB5-infected BALB/c mice in vivo (Figure 1G). In addition, treating SH-SY5Y cells with the inflammatory mediators (TNF-α and IL-1β) and LPS significantly increased the expression of LINC1467 (Figure 1H).

In summary, the expression of LINC1467 is primarily induced by viral genomic RNA and inflammatory stimuli, with broad expression across multiple cell lines and tissues. These findings suggest LINC1467 may play a critical role in host innate immune responses during HFMD-related EV infection.

### 3.2. LINC1467 Suppresses HFMD Pathogen Replication by Regulating the NF-κB Pathway

**Figure 2:**
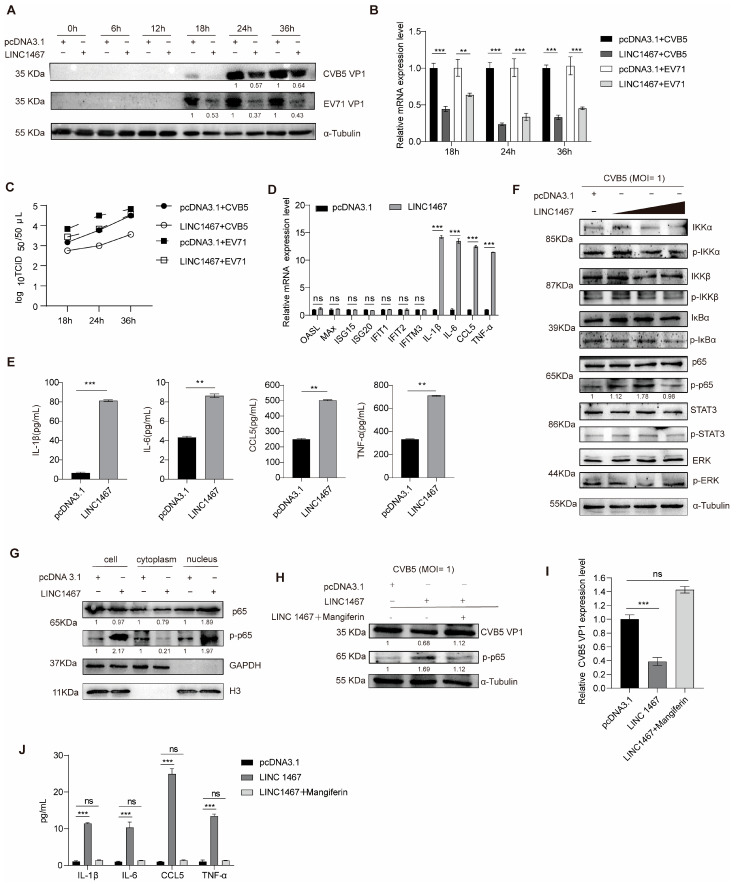
LINC1467 inhibits HFMD pathogen replication via p65. SH-SY5Y cells were transfected with LINC1467 overexpressing plasmid (LINC1467) or an empty vector (pcDNA3.1), followed by infection with CVB5 or EV71 (MOI = 1) at 24 h post-transfection. Cells and supernatants were harvested at 6, 12, 18 24, or 36 h post-infection (hpi). (**A**) The expression of VP1 protein was measured by Western blotting; (**B**) the expression of VP1 mRNA was measured by RT-qPCR; (**C**) viral titers were measured by a TCID50 assay; (**D**) the expression of ISGs and inflammatory factors mRNA were measured by RT-qPCR; (**E**) the secretion of inflammatory factors was measured by ELISA; (**F**) the expression of the inflammation key proteins was measured by Western blotting; (**G**) the levels of p65/p-p65 in cytoplasm and nucleus were measured by Western blotting; (**H**–**J**) mangiferin was added to SH-SY5Y cells. The expression of CVB5 VP1 was measured by Western blotting (**H**) and RT-qPCR (**I**); the expression of inflammatory factors mRNA was measured by RT-qPCR (**J**). All data are presented as the mean ± SD from biologically independent experiments (*n* = 3). Significant analysis was performed using Student’s *t*-test: *p* ≤ 0.01 (**), *p* ≤ 0.001 (***) and ns for no significant difference. The band intensity of proteins was quantified, and the ratios of the target protein to α-Tubulin are shown.

To investigate the functional role of LINC1467 during infection with HFMD-related EVs, we successfully constructed the LINC1467 overexpression plasmid with pcDNA3.1 vector (LINC1467), and designed ASOs to knock down its expression (ASO-LINC1467) in SH-SY5Y cells (Figure S4). The overexpression of LINC1467 in SH-SY5Y cells, followed by infection with either CVB5, EV71, or CVA16. LINC1467 significantly reduced both VP1 protein and mRNA levels (Figure 2A,B and Figure S5). Additionally, the TCID_50_ assay confirmed that LINC1467 significantly reduced viral titers (Figure 2C and Figure S5). In contrast, the knockdown of LINC1467 markedly promoted viral replication (Figures S5 and S6). Importantly, a CellTiter-Glo assay confirmed that LINC1467 had no impact on SH-SY5Y cell viability (Figure S7). Collectively, these findings indicate that LINC1467 suppresses the replication of HFMD-related EVs.

To explore the role of LINC1467 in regulating inflammatory responses, both RT-qPCR and ELISA analyses showed that LINC1467 significantly increased the levels of the pro-inflammatory cytokines (TNFα, IL-6, IL-1β, and CCL5), without significantly affecting the expression of interferon-stimulated genes (ISGs) (Figure 2D,E). Conversely, LINC1467 knockdown reduced the levels of these pro-inflammatory cytokines, while leaving ISG expression unchanged (Figure S8). We next investigated the impact of LINC1467 on the key inflammation pathway during viral infection. We observed that LINC1467 significantly promoted the phosphorylation of the NF-κB subunit p65 (Figure 2F) and enhanced the nuclear translocation of phosphorylated p65 (p-p65) (Figure 2G). Conversely, LINC1467 knockdown inhibited p65 phosphorylation (Figure S8C). To determine whether the antiviral function of LINC1467 depends on p-p65 nuclear translocation, SH-SY5Y cells were transfected with LINC1467 and then treated with mangiferin, an inhibitor of p65 nuclear translocation. Mangiferin treatment effectively abolished both the antiviral effect and the pro-inflammatory cytokine upregulation induced by LINC1467 (Figure 2H,I), indicating that the nuclear translocation of p-p65 is essential for LINC1467 function.

Together, these results demonstrate that LINC1467 exerts its antiviral effects by promoting p65 phosphorylation and nuclear translocation, thereby activating the NF-κB pathway and enhancing pro-inflammatory responses.

### 3.3. LINC1467 Specifically Interacts with the Host Protein IPO8

**Figure 3:**
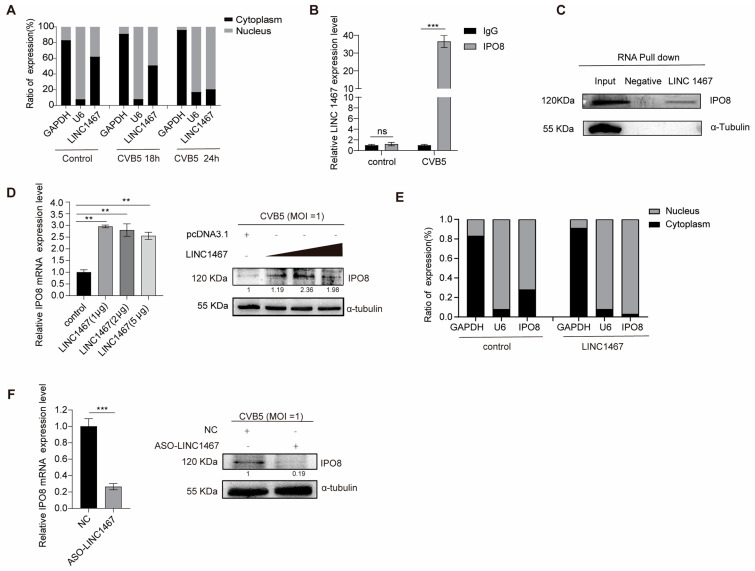
LINC1467 binds to IPO8. (**A**) SH-SY5Y cells infected with CVB5 were fractionated. The expression of LINC1467 in different subcellular fractions was measured by RT-qPCR. GAPDH and U6 mRNA were used as cytoplasmic and nuclear controls, respectively; (**B**) SH-SY5Y cells were infected with CVB5 (MOI = 1) and harvested at 24 hpi. RIP was performed using an anti-IPO8 antibody (IgG as control), and LINC1467 enrichment was measured by RT-qPCR (% input); (**C**) SH-SY5Y cells were infected with CVB5 (MOI = 1) and harvested at 24 hpi. RNA pull-down assay analysis of biotinylated LINC1467 binding to IPO8 by Western blotting; (**D**) SH-SY5Y cells were transfected with LINC1467 or an empty vector (pcDNA3.1), followed by infection with CVB5 (MOI = 1) at 24 h post-transfection. Cells were harvested at 24 hpi. The expression of IPO8 was measured by RT-qPCR and Western blotting; (**E**) SH-SY5Y cells were transfected with LINC1467 or an empty vector (pcDNA3.1) for 24 h, followed by subcellular fractionation. The expression of IPO8 in the cytoplasm and nucleus was measured by RT-qPCR. (**F**) SH-SY5Y cells were transfected with ASO-LINC1467 or a negative control (NC), followed by infection with CVB5 (MOI = 1) at 24 h post-transfection. The expression of IPO8 was measured by RT-qPCR and Western blotting. All data are presented as the mean ± SD from biologically independent experiments (*n* = 3). Significant analysis was performed using Student’s *t*-test: *p* ≤ 0.01 (**), *p* ≤ 0.001 (***), and ns for no significant difference. The band intensity of proteins was quantified, and the ratios of the target protein to α-Tubulin were shown.

To investigate the mechanism by which LINC1467 regulates p65, we first assessed its subcellular localization during CVB5 infection. The results demonstrated that increasing the duration of CVB5 infection significantly elevated the nuclear proportion of LINC1467 (Figure 3A), suggesting that LINC1467 translocates to the nucleus post-infection to exert its regulatory functions. To determine whether LINC1467 functions via epigenetic modulation, we performed RIP assays. However, no direct interaction was observed between LINC1467 and histone H3 (Figure S9A). Similarly, RIP analysis confirmed that LINC1467 does not directly bind to p65 (Figure S9B). These findings hypothesize that LINC1467 may regulate p65 indirectly through interaction with a host regulatory protein.

To identify potential LINC1467-binding proteins, we conducted RNA pull-down assays followed by SDS-PAGE and silver staining. A distinct protein band within the 100–130 kDa range was specifically enriched in the LINC1467 pull-down sample (Figure S10). The mass spectrometry analysis of this band, combined with bioinformatic analysis, predicted several potential interactors, including IPO8, XPO1, TLR3, and TERT (Table 2). Given previous reports linking Importin 8 (IPO8) to p65 nuclear translocation, IPO8 was selected for further validation. Subsequently, both RIP and RNA pull-down assays confirmed a specific interaction between LINC1467 and IPO8 (Figure 3B,C). To explore the functional significance of their interaction, LINC1467 overexpressed in SH-SY5Y cells followed by CVB5 infection. The results demonstrated that LINC1467 upregulated both mRNA and protein levels of IPO8 (Figure 3D). Additionally, subcellular fractionation showed that LINC1467 overexpression significantly increased the nuclear abundance of IPO8 (Figure 3E). In contrast, LINC1467 knockdown suppressed IPO8 expression at both the mRNA and protein levels (Figure 3F). Notably, CVB5 infection alone did not significantly alter IPO8 expression (Figure S11).

These findings indicate that LINC1467 directly binds to IPO8 and enhances its expression and nuclear accumulation, implicating IPO8 as a key mediator through which LINC1467 regulates the NF-κB pathway during viral infection.

### 3.4. Functional Characterization of IPO8

**Figure 4:**
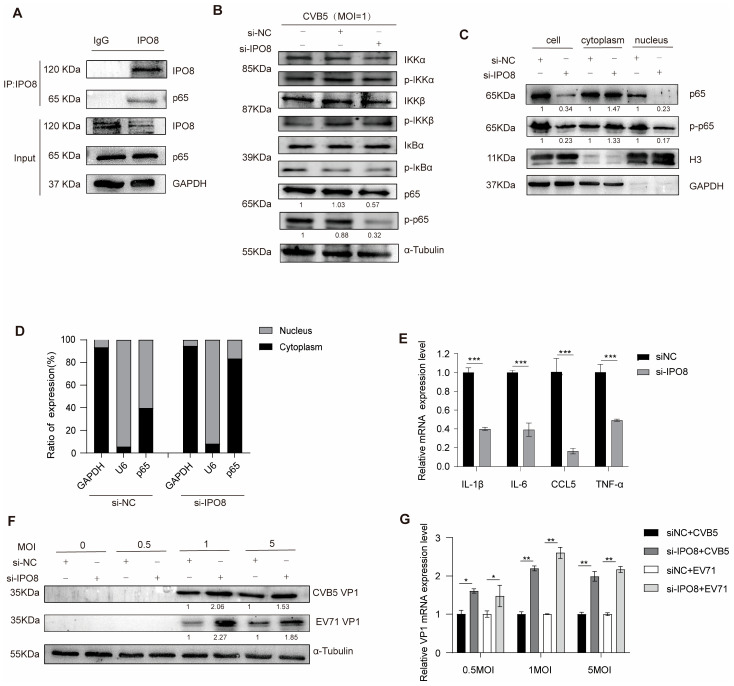
Functional characterization of IPO8. (**A**) HEK293T cells were co-transfected with pcDNA3.1-IPO8 and pcDNA3.1-p65 for 24 h. Co-IP analysis was performed to determine the interaction between IPO8 and p65; (**B**–**G**) SH-SY5Y cells were transfected with si-IPO8 or a negative vector (si-NC), followed by CVB5 infection at 24 h post-transfection. Cells were harvested at 24 hpi. The expression of proteins was measured by Western blotting (**B**); the expression of p65/p-p65 proteins in the cytoplasm and nucleus was measured by Western blotting (**C**) and RT-qPCR (**D**). GAPDH and U6 mRNA were, respectively, used as cytoplasmic and nuclear controls; the expression of inflammatory factors mRNA was measured by RT-qPCR (**E**); the expression of VP1 was measured by Western blotting (**F**) and RT-qPCR (**G**). All data are presented as the mean ± SD from biologically independent experiments (*n* = 3). Significant analysis was performed using Student’s *t*-test: *p* ≤ 0.05 (*), *p* ≤ 0.01 (**), *p* ≤ 0.001 (***), and ns for no significant difference. The band intensity of proteins was quantified, and the ratios of the target protein to α-tubulin are shown.

To explore the functional role of IPO8, we first confirmed the interaction between IPO8 and p65 via co-immunoprecipitation (Co-IP) assays (Figure 4A). Next, IPO8 was knocked down in SH-SY5Y cells, followed by CVB5 infection. Western blotting analysis revealed that IPO8 knockdown specifically reduced the levels of both total p65 and its phosphorylated form (p-p65), without significantly affecting other key components of the NF-κB pathway (Figure 4B). Subcellular fractionation analysis further demonstrated that IPO8 knockdown significantly impaired the nuclear translocation of p65 (Figure 4C,D). Consistently, RT-qPCR analysis showed that the knockdown of IPO8 reduced the mRNA expression of NF-κB-dependent pro-inflammatory cytokines (Figure 4E). These results suggest that IPO8 facilitates p65 activation and nuclear import, thereby promoting NF-κB-mediated inflammatory responses. We next examined the antiviral role of IPO8 during HFMD-related EV infection. Western blotting and RT-qPCR analyses revealed that he knockdown of IPO8 significantly enhanced both the VP1 protein and mRNA levels of CVB5 and EV71 (Figure 4F,G). This finding indicates that IPO8 contributes to the host antiviral defense by enhancing p65-mediated immune signaling and inhibiting viral replication.

### 3.5. LINC1467 Exerts Its Function in an IPO8-Dependent Manner

**Figure 5:**
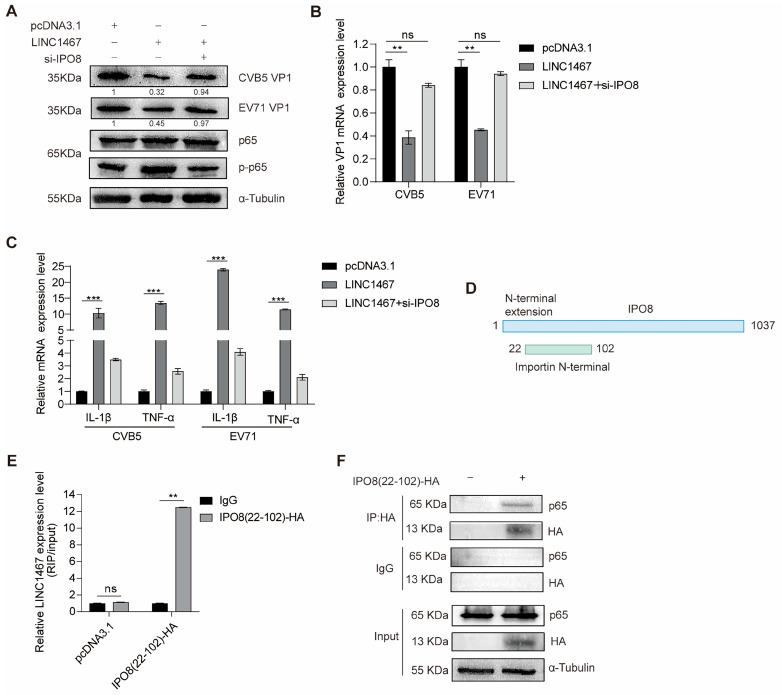
LINC1467 relies on IPO8 to exert effects. (**A**–**C**) SH-SY5Y cells were co-transfected with LINC1467 and/or si-IPO8, followed by infection with CVB5 or EV71 (MOI = 1) at 24 h post-transfection. Cells were harvested at 24 hpi. The expression of p65 and VP1 was measured by Western blotting (**A**); the expression of VP1 mRNA was measured by RT-qPCR (**B**); the expression of inflammatory factors mRNA was measured by RT-qPCR (**C**); (**D**) schematic representation of the IPO8 protein structure, highlighting the 22–102 amino acid (AA) domain; (**E**) SH-SY5Y cells were infected with CVB5 (MOI = 1) and harvested at 24 hpi. The binding between LINC1467 and IPO8 22–102 domain was confirmed by RIP-qPCR (%input); (**F**) HEK293T cells were transfected with IPO8 22–102, and the Co-IP analysis was performed to determine the interaction between IPO8 22–102 and p65. All data are presented as the mean ± SD from biologically independent experiments (*n* = 3). Significant analysis was performed using Student’s *t*-test: *p* ≤ 0.01 (**), *p* ≤ 0.001 (***), and ns for no significant difference. The band intensity of proteins was quantified, and the ratios of the target protein to α-tubulin are shown.

To determine whether the antiviral effects of LINC1467 depend on IPO8, SH-SY5Y cells were co-transfected with the following plasmids, followed by infection with CVB5 or EV71. Compared to cells transfected with LINC1467 alone, IPO8 knockdown effectively reversed the LINC1467-induced phosphorylation of p65 (Figure 5A), abolished the inhibitory effect of LINC1467 on viral replication (Figure 5B), and suppressed the LINC1467-mediated upregulation of pro-inflammatory cytokines (Figure 5C). These findings indicate that IPO8 is essential for the antiviral and immunomodulatory functions of LINC1467.

Bioinformatic analysis predicted that IPO8 comprises 1037 amino acid (AA) protein, with a conserved functional domain spanning residues 22–102 (Figure 5D). Further prediction suggested that both LINC1467 and p65 may interact with this specific domain (22-102AA). Subsequent RIP assays confirmed that LINC1467 is specifically bound to the 22-102AA domain of IPO8 (Figure 5E), while Co-IP assays verified that p65 also interacts with the same domain (Figure 5F). Together, these results suggest that LINC1467 and p65 co-occupy the 22–102 AA domain of IPO8, supporting a model in which LINC1467 facilitates p65 activation and nuclear translocation through its interaction with IPO8. IPO8 has critical role as a molecular bridge linking LINC1467 to the NF-κB pathway.

### 3.6. Validation of the Antiviral Role of LINC1467 in Vivo

**Figure 6:**
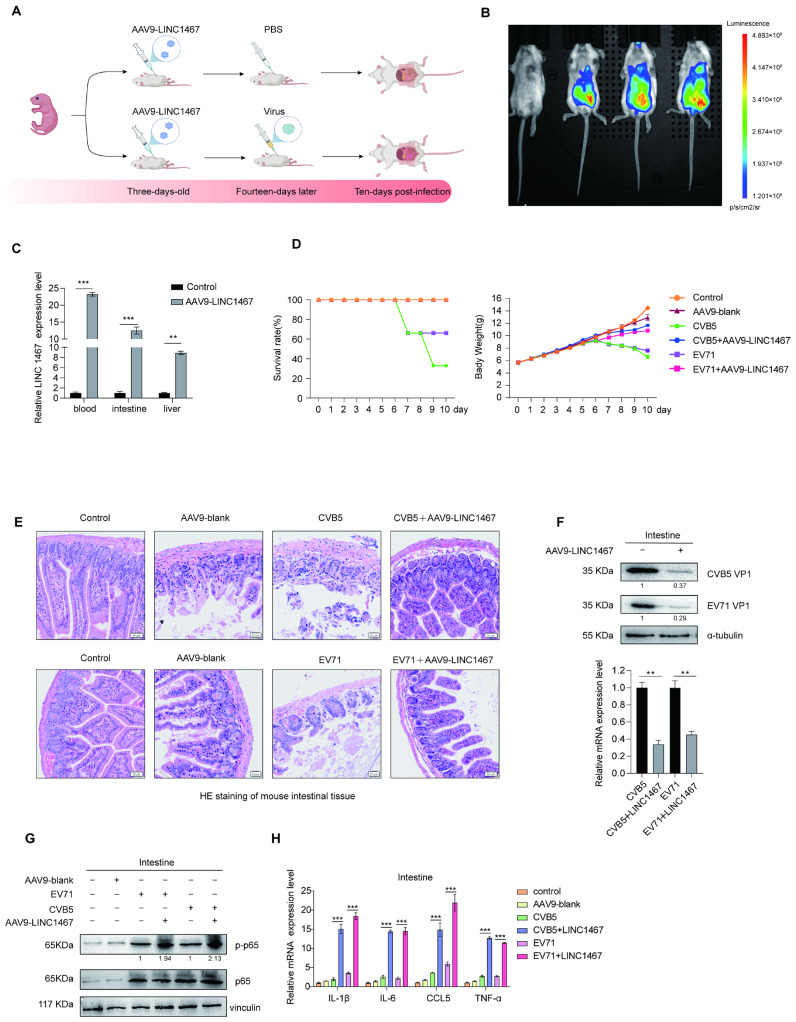
The antiviral role of LINC1467 in vivo. (**A**) Schematic of the mouse infection model. Three-day-old BALB/c mice were injected intraperitoneally with AAV9-LINC1467 (5 × 10^11^ Genomic Copies) or AAV9 (2 × 10^11^ Genomic Copies) three times. Fourteen days later, the mice were infected with CVB5 or EV71 (20 LD50) by intraperitoneal injection. Samples were collected ten days post-infection; (**B**) the expression of LINC1467 in mice was detected by bioluminescence imaging; (**C**) the expression of LINC1467 was detected by RT-qPCR; (**D**) mouse survival rates and body weights were recorded and statistically analyzed; (**E**) pathological changes in intestines were assessed by HE staining; (**F**) the expression of VP1 in intestines was measured by Western blotting and RT-qPCR; (**G**) the expression of p65/p-p65 was measured by Western blotting; (**H**) the expression of inflammatory factors in the intestines was measured by RT-qPCR. Six BALB/c mice were used for the experimental group. All data are presented as the mean ± SD from biologically independent experiments (*n* = 3). Significant analysis was performed using Student’s *t*-test: *p* ≤ 0.01 (**), *p* ≤ 0.001 (***). The band intensity of the proteins was quantified, and the ratios of the target protein to α-tubulin are shown.

To validate the antiviral function of LINC1467 in vivo, we established the viral infection model in BALB/c mice with LINC1467 overexpression (Figure 6A). Three-day-old mice were intraperitoneally injected with AAV9-LINC1467. Successful overexpression was confirmed via bioluminescence imaging and RT-qPCR analysis, indicating the stable expression of LINC1467 in the mouse model (Figure 6B,C). Subsequently, mice were challenged with either CVB5 or EV71 via intraperitoneal injection. Ten days post-infection, body weight and survival rates were recorded, and the intestine was collected for analysis. Compared to the control group, the LINC1467 overexpression group significantly improved the survival rate and less weight loss (Figure 6D). HE staining further confirmed that LINC1467 overexpression markedly alleviated intestinal tissue damage caused by viral infection (Figure 6E), suggesting its protective effect against HFMD-related EV-induced pathology. Furthermore, in the intestinal tissue, LINC1467 overexpression significantly decreased VP1 protein and mRNA levels for both CVB5 and EV71 (Figure 6F). Concurrently, it enhanced the expression of p-p65 and downstream pro-inflammatory cytokines (Figure 6G,H). All the in vivo results confirm that LINC1467 effectively inhibits HFMD-related EVs, and attenuates virus-induced tissue damage, highlighting its potential as a therapeutic target for antiviral.

## 4. Discussion

EV71, CVA16, and CVB5 are major causative agents of HFMD. Although lncRNA profiling has been conducted in cells infected with these enteroviruses [16,17,18], the regulatory mechanisms and function of specific lncRNAs remain largely unclear. Previously, our lab identified two lncRNAs involved in CVB5 infection, such as LINC1392 which inhibited the replication of CVB5 by regulating the melanoma differentiation associated gene 5 (MDA5) via ELAV-like RNA binding protein 1 (ELAVL1) [19]. Additionally, LINC2781 activated the JAK-STAT pathway and blocked G3BP2-mediated STAT1 degradation, enhancing antiviral immune against CVB5 infection [20]. In our present research, we identified a novel lncRNA, LINC1467, which was highly inducible upon CVB5 and EV71 infection in SH-SY5Y cells. LINC1467 is a 457-nucleotide intergenic lncRNA located on chromosome 2, and lacks protein-coding potential, consistent with the characteristics of most lncRNAs. Among numerous lncRNAs induced during viral infection, LINC1467 exhibited time- and dose-dependent effects on EV71 and CVB5 infection. Its expression was specifically triggered by viral genomic RNA, rather than by non-structural viral proteins, aligning with the response to poly(I:C) stimulation. Such induction by viral genome components has been rarely reported, suggesting a potentially novel mechanism of lncRNA activation. Furthermore, LINC1467 was found to be evolutionarily conserved across species and broadly expressed across multiple cell types and tissues, indicating a non-type-specific regulatory role. Collectively, these findings suggest that LINC1467 plays an important role in the host response to HFMD-related viruses.

Functionally, LINC1467 was shown to suppress the replication of EV71, CVA16, and CVB5. Notably, as the incidence of co-infections and novel recombinant enteroviruses in HFMD cases has increased in recent years [21,22], the ability of LINC1467 effectively inhibit multiple EVs highlights its potential as a broad-spectrum antiviral factor and a promising candidate. Transcriptomic and cytokine profiling revealed that LINC1467 enhances the expression of pro-inflammatory cytokines such as TNF-α, IL-6, IL-1β, and CCL5. Further pathway analysis showed that this effect was mediated through selective activation of the NF-κB pathway, specifically via phosphorylation and nuclear translocation of the p65 subunit, with no significant effect observed on other inflammation-related pathways such as MAPK or JAK-STAT3 [23,24,25]. Importantly, treatment with mangiferin, an inhibitor of p65 nuclear translocation [26], confirmed the dependence on NF-κB signaling. These results showed that LINC1467 promotes p65 phosphorylation and subsequent nuclear translocation, thereby activating the NF-κB pathway and promoting the expression of downstream inflammatory cytokines that exert antiviral effects. NF-κB is a crucial regulator of the innate immune response and comprises five members (p65/RelA, RelB, c-Rel, p50, and p52) that coordinate inflammatory signaling [27]. Upon RNA viral infection, cellular receptors recognize viral components and initiate signaling cascades that lead to p65 phosphorylation and nuclear translocation [28]. Many lncRNAs have been reported to modulate NF-κB activity by directly interacting with its components [29,30]; however, our study identifies an alternative mechanism in which LINC1467 does not bind directly to p65, but instead regulates its nuclear translocation indirectly via protein intermediaries.

The subcellular localization of lncRNAs often dictates their function. Cytoplasmic lncRNAs typically modulate mRNA stability or translation [31,32,33], whereas nuclear lncRNAs regulate transcription, chromatin accessibility, or RNA processing. For example, NRAV suppresses ISG transcription by altering histone modifications, thereby facilitating viral replication [34]. In our study, LINC1467 was predominantly localized to the nucleus, but did not associate with histone H3, suggesting a non-epigenetic mechanism. Instead, we identified IPO8 as a direct binding partner of LINC1467. IPO8, an important member of the karyopherin β family, functions as a key transporter of macromolecules between the cytoplasm and nucleus in mammalian cells. These transporters recognize nuclear localization or export signals on cargo molecules, enabling their transport between the cytoplasm and nucleus [35]. Previous studies have reported that IPO8 plays a critical role in mediating the cytoplasm-to-nucleus transport of mature miRNAs [36], and have been linked to disorders involving immune dysregulation, cardiovascular defects, and skeletal abnormalities [37]. However, its role in viral infections remains largely unexplored. Our findings demonstrate that IPO8 directly interacts with both LINC1467 and p65, enhancing p65 phosphorylation and nuclear import, thereby enhancing NF-κB activation, consistent with earlier reports [38]. Structural mapping revealed that LINC1467 and p65 both bind to the same functional domain (amino acids 22–102) of IPO8, supporting a model in which LINC1467 functions as a molecular scaffold, facilitating the formation of a LINC1467/IPO8/p65 complex that enhances innate immune activation.

**Figure 7:**
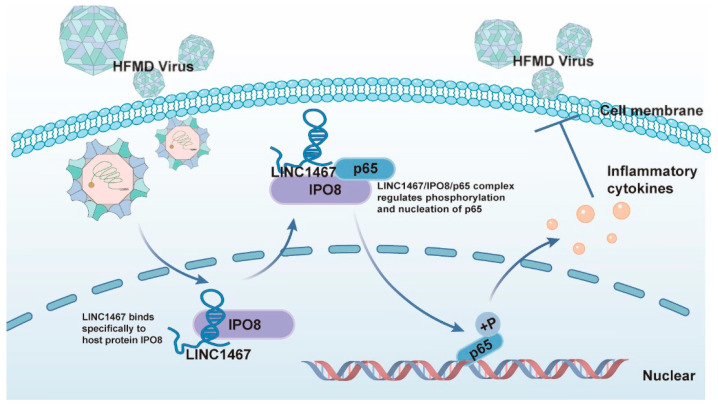
A proposed model for the LINC1467 restriction HFMD virus replication via the IPO8–p65 axis.

In conclusion, we identified a previously uncharacterized lncRNA, LINC1467, as a critical antiviral regulator in the host response to HFMD-related EV infection. LINC1467 promotes innate immunity by directly interacting with the nuclear import receptor IPO8, forming a tripartite complex with p65, and facilitating its phosphorylation and nuclear translocation. This activation of the NF-κB signaling cascade promotes the expression of pro-inflammatory cytokines, thereby enhancing the host antiviral defense (Figure 7). These findings provide new insights into lncRNA-mediated antiviral mechanisms and identify LINC1467 as a promising target for therapeutic intervention against enteroviral infections.

## Figures and Tables

**Table 1:** Primer sequences for RT-qPCR.

Gene	Forward Primer (5′–3′)	Reverse Primer (5′–3′)
CVB5-VP1	CCAGTGCCCACGAAATAAA	TTGCCTATGCTGATGAACGGT
EV71-VP1	GCAGCGGAACCGACTACTTTG	GCCTGYCTAAGRCCTGCGAA
Human-LINC1467	CTGCCCTTGACACCTCAAAGA	AACAGAGGCAAACAGTCTCCA
Mouse-LINC1467	CGGTGCAGGAGAATCTGTCA	AACAGAGGCAAACAGTCTCCA
TNF-α	GCCACCACGCTCTTCTGTCTAC	GGGTCTGGGCCATAGAACTGAT
IL-1β	ACCTTCCAGGATGAGGACATGA	CTAATGGGAACGTCACACACCA
IL-6	CACATGTTCTCTGGGAAATCG	TTGTATCTCTGGAAGTTTCAGAT
CCL5	CGCTGTCATCCTCATTGCTA	CCATTTCTTCTGGGTT
OASL	TTGTGCCTGCCTACAGAGC	TTCAGCTTAGTTGGCCGATGT
MXA	TTCAGCACCTGATGGCCTATC	TGGATGATCAAAGGGATGTGG
ISG15	CTCTGAGCATCCTGGTGAGGAA	AAGGTCAGCCAGAACAGGTCGT
ISG20	TGACCTGAAGCACGACTTCC	CAGGCTGTTCTGGATGCTCT
IFIT1	TCTCAGAGGAGCCTGGCTAAG	CCACACTGTATTTGGTGTCTAGG
IFIT2	ACCTCTGGACTGGCAATAGC	GTCAGGATTCAGCCGAATGG
IFITM3	CATCGTCATCCCAGTGCTGAT	ATGGAAGTTGGAGTACGTGGG
IPO8	TGTTCAGCTCCTTCCTGATTC	CTTCTTACACTTCCACCATAC
GAPDH	GTATGACAACGAATTTGGCTACA	AGCACAGGGTACTTTATTGATGG
U6	CTCGCTTCGGCAGCACA	AACGCTTCACGAATTTGCGT

**Table 2:** Proteins binding to LINC1467.

Name	Interaction Propensity	Z-Score	kDa
A2RTX5 TARS3	31.96	1.22	92
A5YKK6 CNOT1	27.54	0.94	266
B5MCY1 TDRD15	29.2	1.05	221
B5ME19 EIF3CL	29.78	1.08	105
O00411 POLRMT	28.42	1	138
O14647 CHD2	29.65	1.08	211
O14746 TERT	29.06	1.04	126
O14776 TCERG1	47.87	2.21	123
O14787 TNPO2	38.15	1.6	101
O14980 XPO1	29.24	1.05	123
O15037 KHNYN	26.76	0.9	74
O15042 U2SURP	38.02	1.6	118
O15381 NVL	34.23	1.36	95
O15397 IPO8	37.06	1.54	120
O15455 TLR3	31.82	1.21	103
O43143 DHX15	27.89	0.97	90
O43290 SART1	50.26	2.36	90

## Data Availability

All data generated in the study are included in this published paper.

## Supplementary Materials

The following supporting information can be downloaded at: https://www.mdpi.com/article/10.3390/pathogens14101071/s1, Figure S1: RT-qPCR validation of the top ten differentially expressed lncRNAs associated with inflammation were validation from the primary RNA-seq results. Figure S2: Prediction of the protein-coding ability of LINC1467 using PhylocSF analysis. Figure S3: Cross-species homology analysis of LINC1467 based on multiple sequence alignment. Figure S4: Validation of successful LINC1467 plasmid and ASO-LINC1467 construction. Figure S5: LINC1467 inhibits the replication of CVA16. Figure S6: LINC1467 inhibits the replication of CVB5 and EV71. Figure S7: LINC1467 does not affect cell viability. Figure S8: LINC1467 activates NF-κB pathway via p65. Figure S9: LINC1467 does not directly bind to histone H3 or p65 protein. Figure S10: Identification of LINC1467-binding proteins by silver staining. Figure S11: The expression of IPO8.
