# Supplementary figures and images for: LINC1467 Activates the IPO8–p65 Axis to Restrict Hand, Foot, and Mouth Disease Virus Replication
Source: Pathogens. 2025 Oct 21;14(10):1071. doi: 10.3390/pathogens14101071 (PMC12566618; doi:10.3390/pathogens14101071)

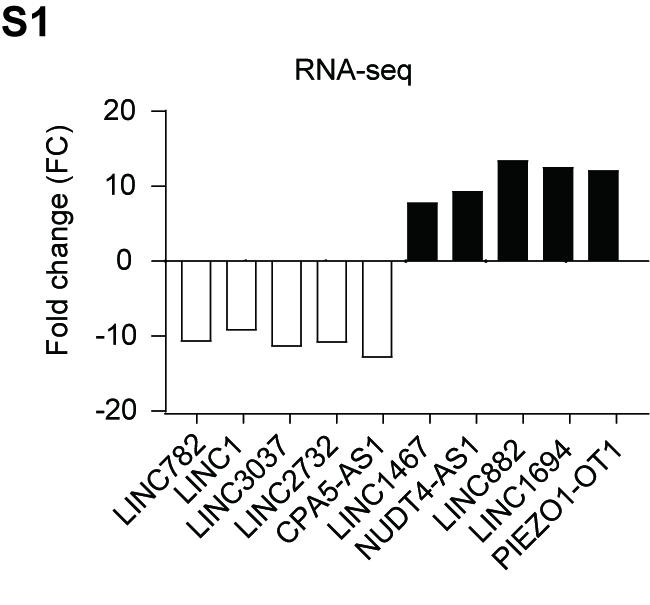

Supplement: Supplementary file 1 [file pathogens-14-01071-s001.zip › Figure S1.tif]

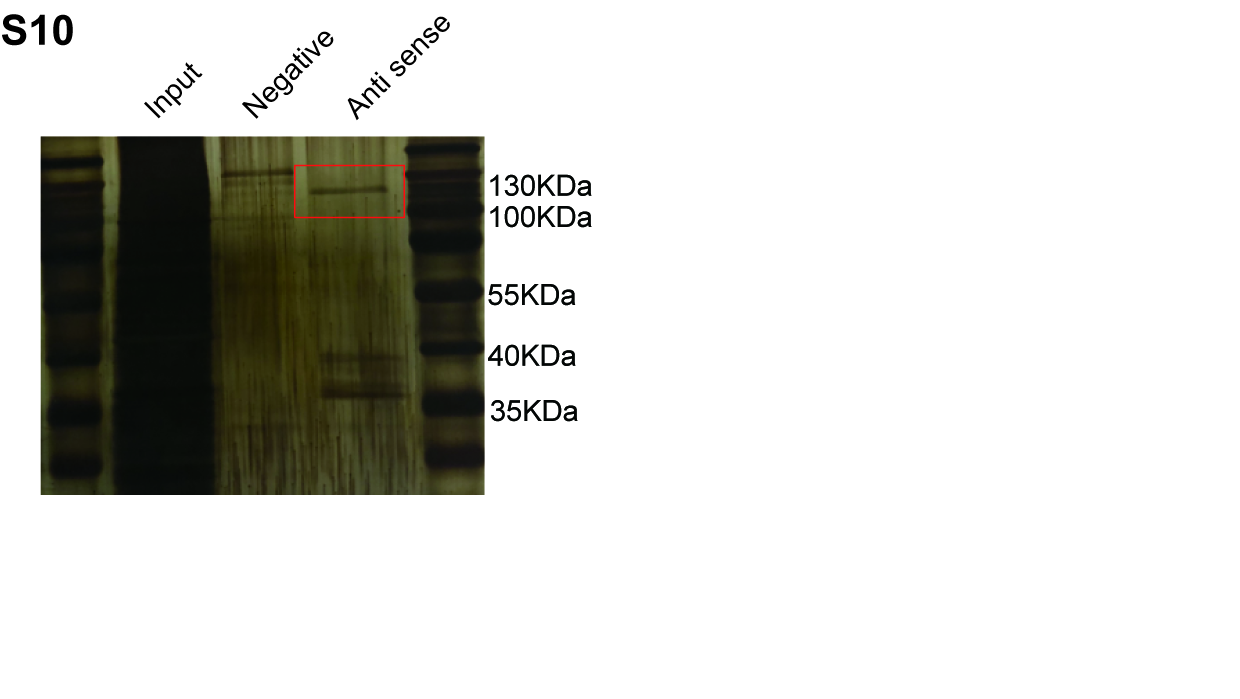

Supplement: Supplementary file 1 [file pathogens-14-01071-s001.zip › Figure S10.tif]

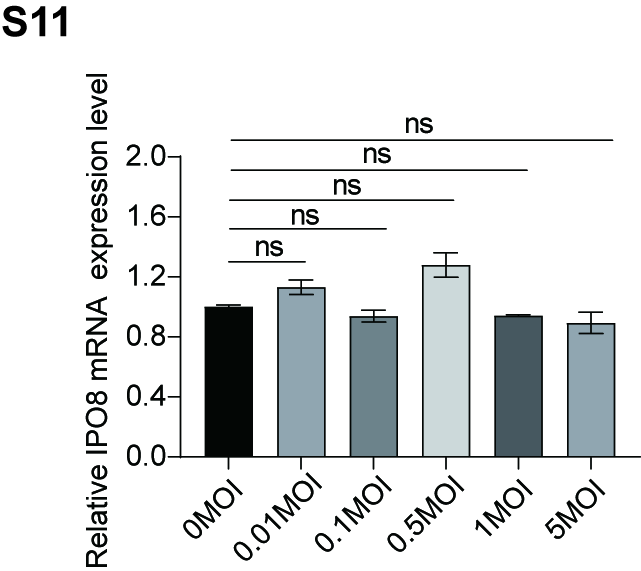

Supplement: Supplementary file 1 [file pathogens-14-01071-s001.zip › Figure S11.tif]

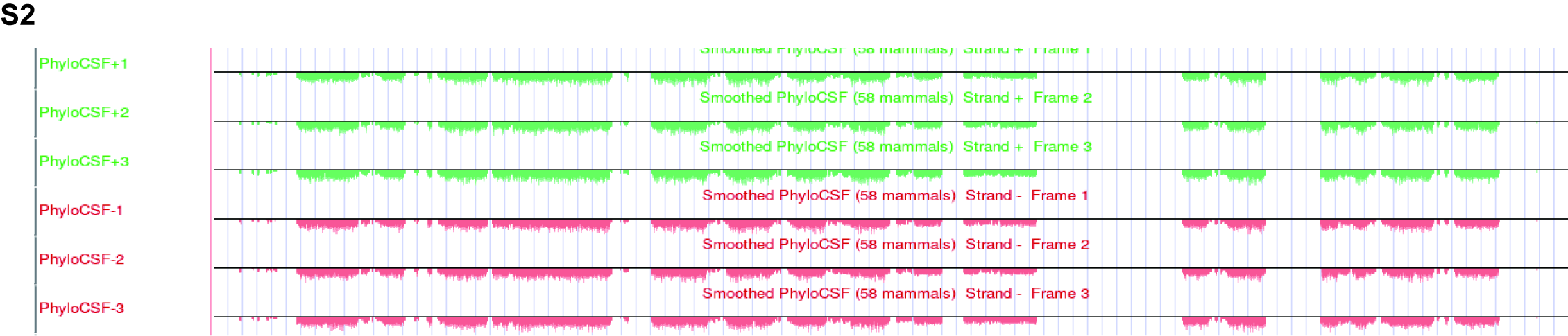

Supplement: Supplementary file 1 [file pathogens-14-01071-s001.zip › Figure S2.tif]

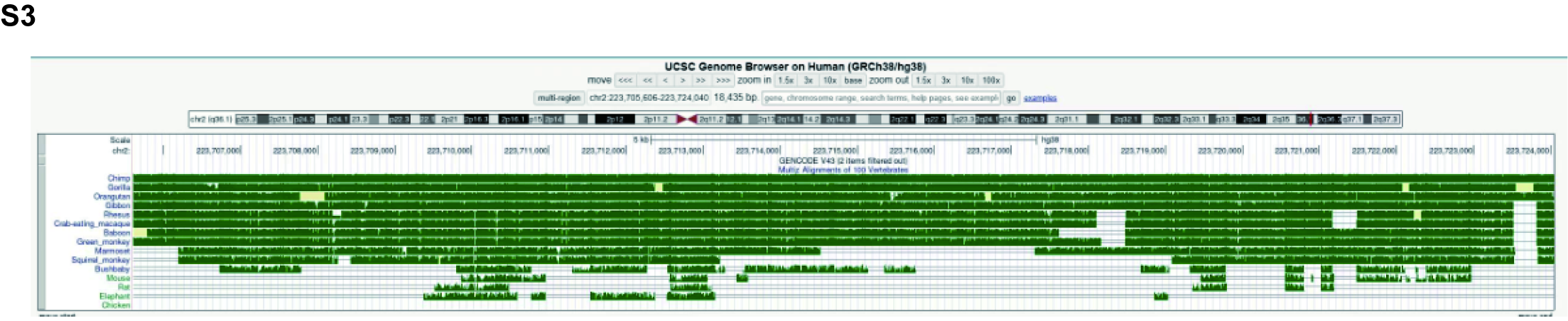

Supplement: Supplementary file 1 [file pathogens-14-01071-s001.zip › Figure S3.tif]

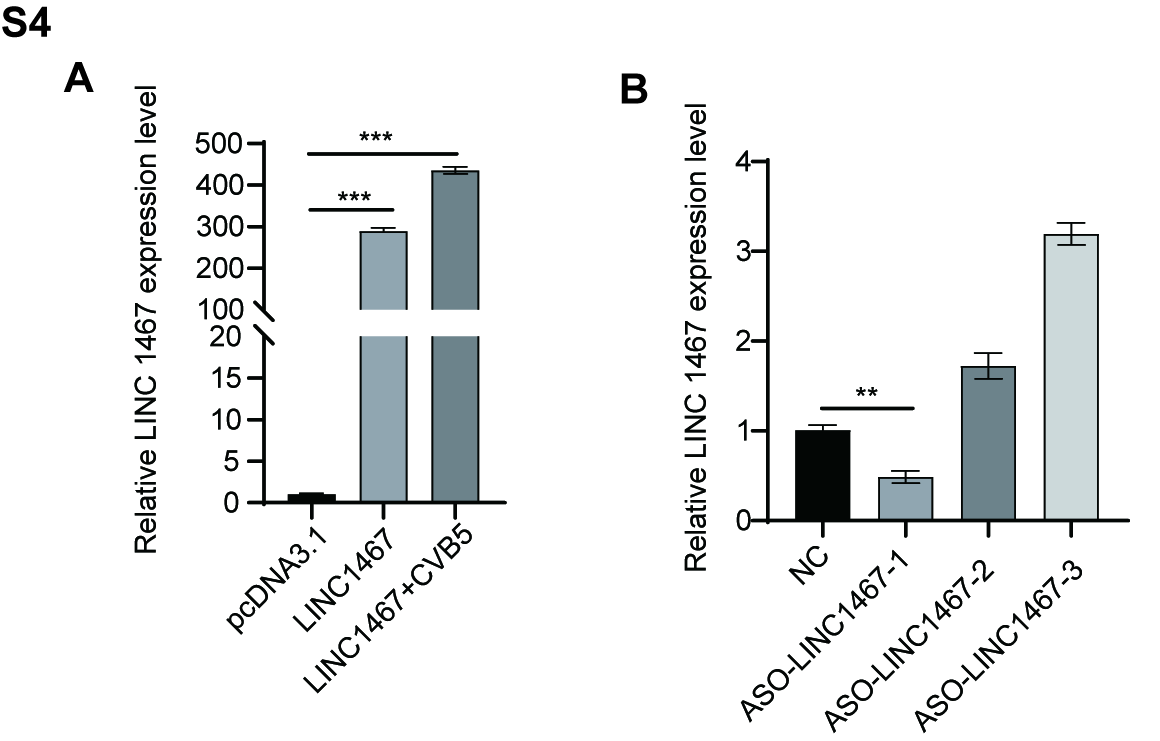

Supplement: Supplementary file 1 [file pathogens-14-01071-s001.zip › Figure S4.tif]

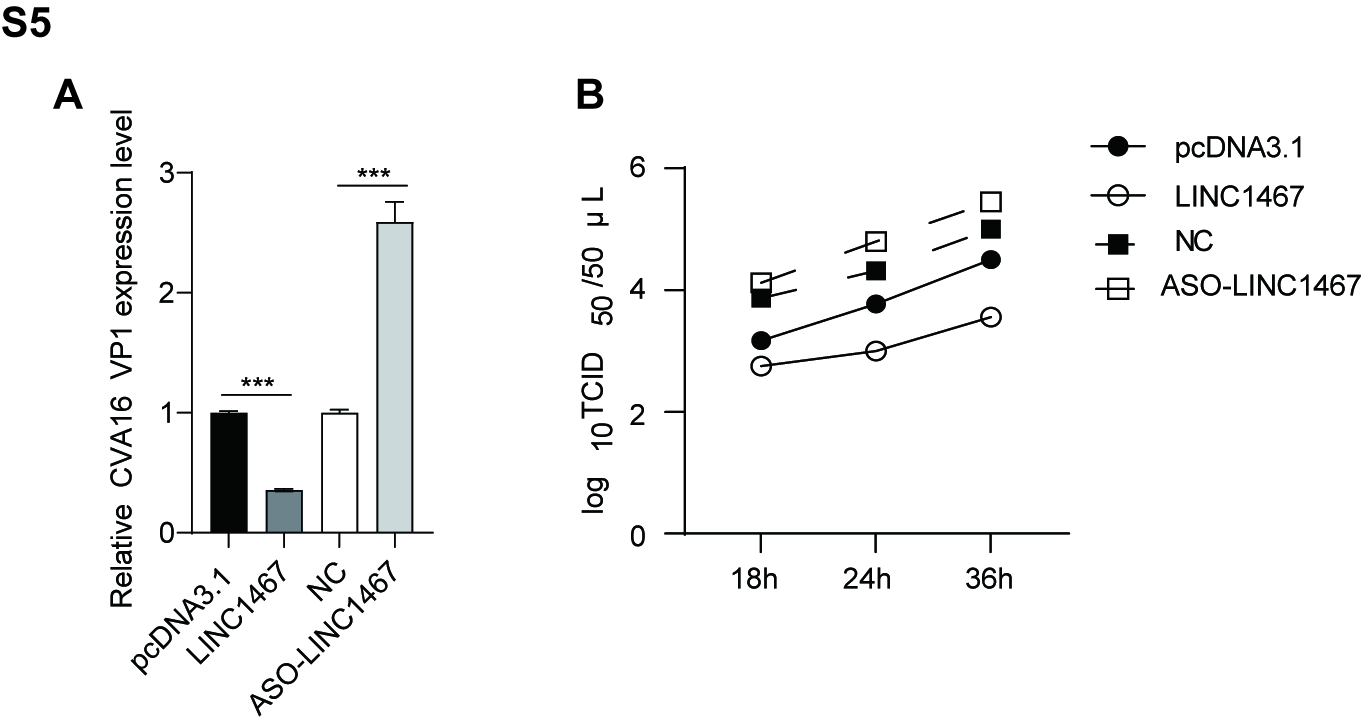

Supplement: Supplementary file 1 [file pathogens-14-01071-s001.zip › Figure S5.tif]

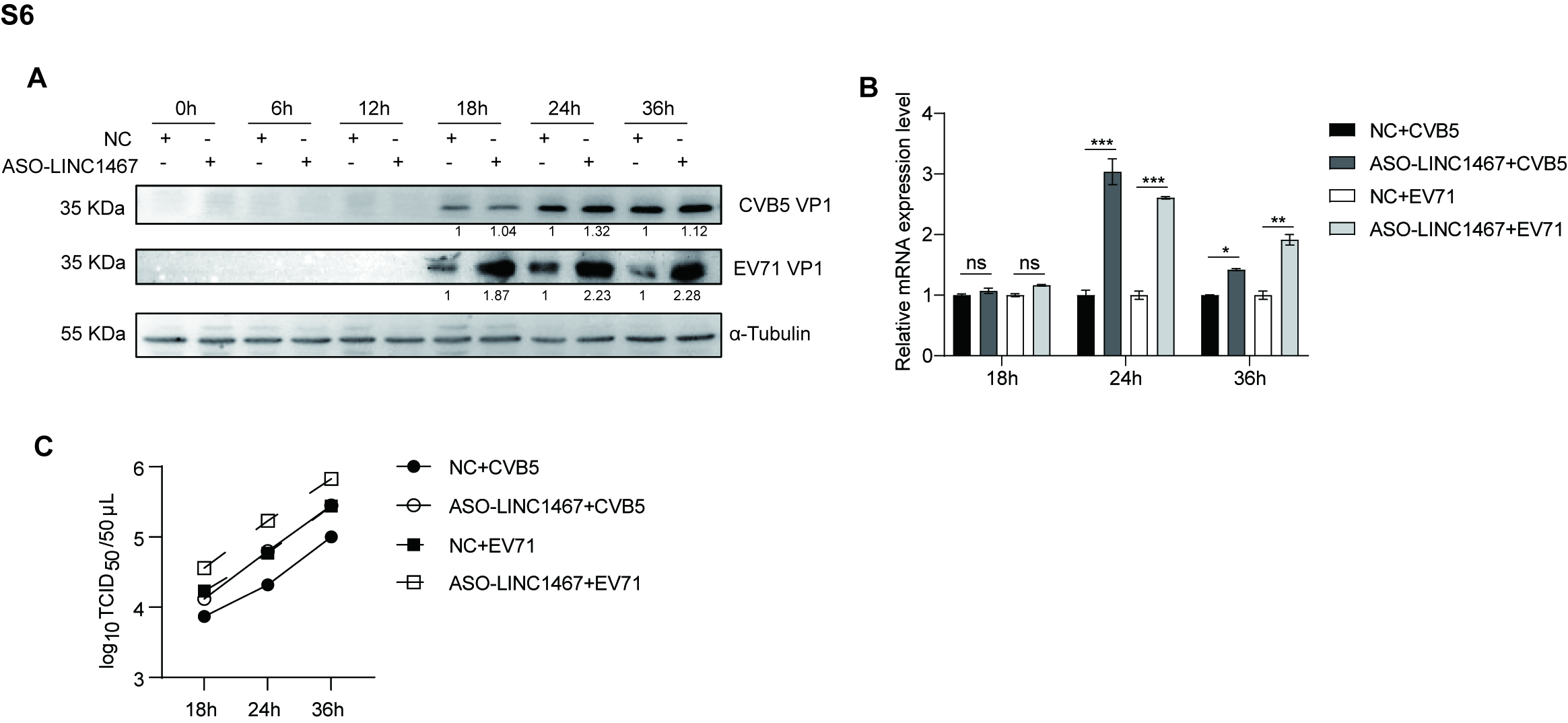

Supplement: Supplementary file 1 [file pathogens-14-01071-s001.zip › Figure S6.tif]

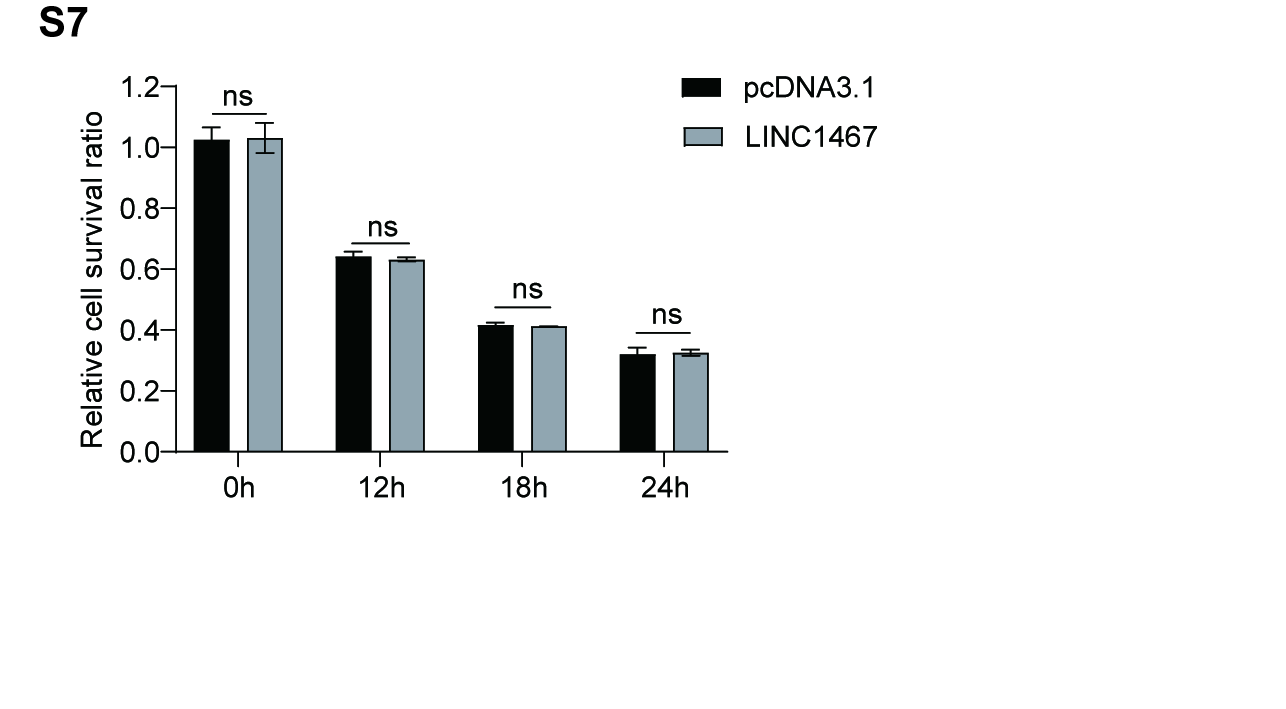

Supplement: Supplementary file 1 [file pathogens-14-01071-s001.zip › Figure S7.tif]

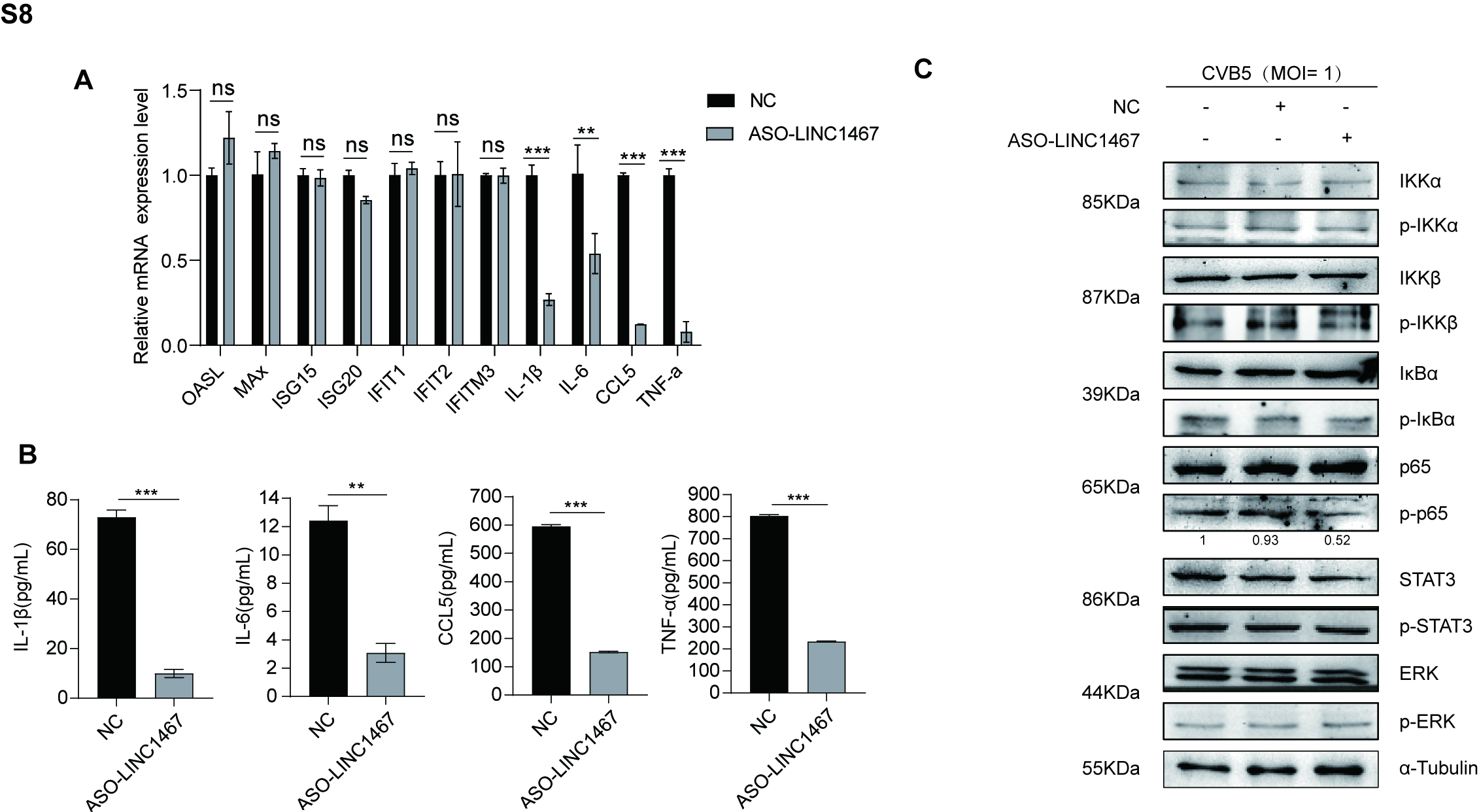

Supplement: Supplementary file 1 [file pathogens-14-01071-s001.zip › Figure S8.tif]

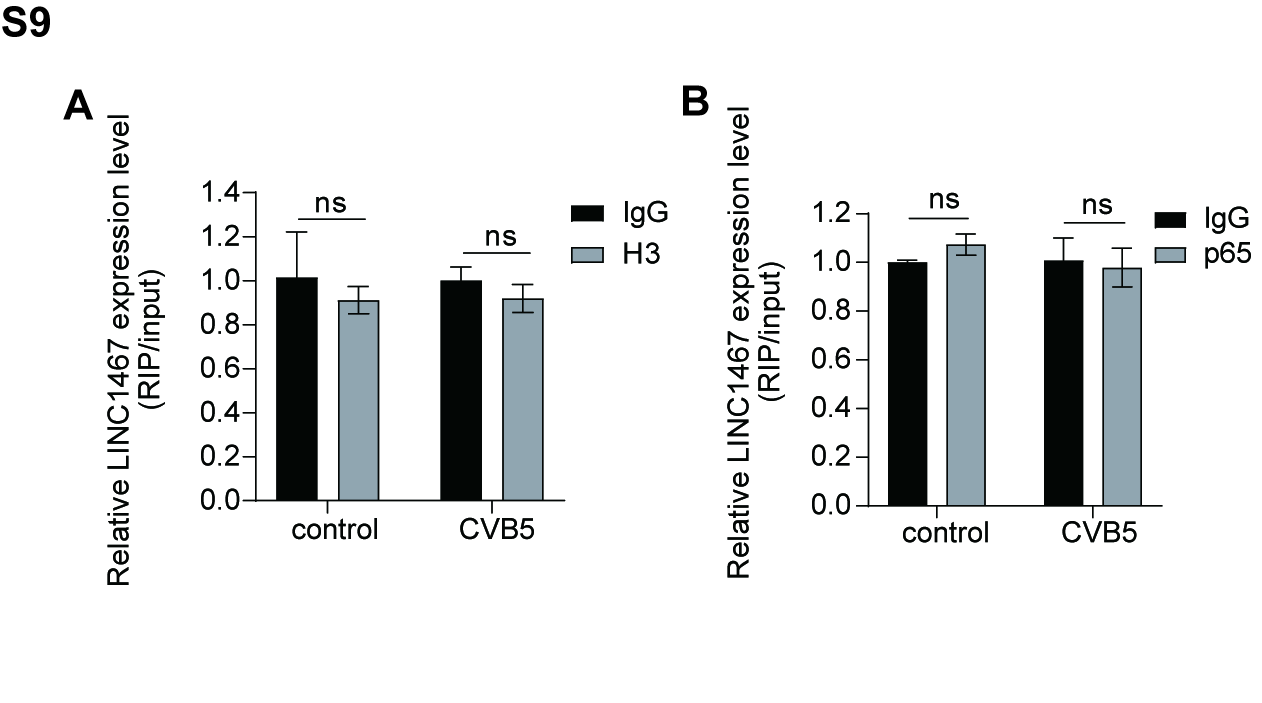

Supplement: Supplementary file 1 [file pathogens-14-01071-s001.zip › Figure S9.tif]
